# Epidemiological and Clinicopathological Trends of Breast Cancer in Chinese Patients During 1993 to 2013: A Retrospective Study

**DOI:** 10.1097/MD.0000000000000820

**Published:** 2015-07-02

**Authors:** Wen Si, Ying Li, Yingjie Han, Fan Zhang, Yingzhe Wang, Ying Li, Rui Xia Linghu, Xingyang Zhang, Junlan Yang

**Affiliations:** From the Department of Medical Oncology I, General Hospital of the Chinese People's Liberation Army, Beijing (WS, YH, FZ, YW, YL, RXL, XZ, JY); School of Medicine, Nankai University, Tianjin (WS, YH); and Department of Medical Oncology, Beijing Geriatric Hospital, Haidian District (YL).

## Abstract

This study aimed to summarize the epidemiological and pathological trends of breast cancer in Chinese women.

The clinical data of 4968 breast cancer patients treated at the Chinese PLA General Hospital from 1993 to 2013 were retrospectively reviewed.

The mean ± standard deviation (SD) age was 47.4 ± 11.3 years before the year 2001, 49.2 ± 11.2 years during 2001 to 2010, and 50.6 ± 11.4 years after the year 2010, respectively (*P* < 0.001). The ratio of premenopausal women to postmenopausal women was 1.6 and no significant changes were found during the period (*P* = 0.121). The proportion of patients with Scarff Bloom Richardson III breast cancer showed significant increase along with time (*P* = 0.015). The breast cancer was accounting for 31.7% at stage I and DCIS/LCIS and tend to be diagnosed with early stage around time (*P* < 0.001). The proportion of DCIS/LCIS and stage I increased with time during the 20 years from 14.6% to 33.2%, whereas the proportion of stage III to IV decreased.

The proportion of Luminal A-like subtype gradually reduced and Luminal B-like (HER2-negative) increased and developed to the predominant type. Older age and earlier stage at diagnosis, as well as the alternation of predominant molecular subtypes, have become the developed trends of breast cancer.

## Abstract 2

Supplemental Digital Content is available in the text

## INTRODUCTION

Breast cancer is the most common malignancy in women worldwide. According to the data from Globocan 2012, the global incidence of breast cancer was estimated at 1.67 million newly diagnosed patients and 0.522 million deaths owing to breast cancer globally (http://globocan.iarc.fr/Pages/fact_sheets_cancer.aspx). Breast cancer is the most important cause of women's cancer related death in less developed regions with 324,000 deaths per year (14.3% of total) and the second cause in more developed regions with 198,000 deaths per year (15.4%). Differences in the incidence, pathology, and mortality of breast cancer have been noticed between different regions and races,^1–5^ which may be related to genetic background, socioeconomic development, life style, and so on.

The burden of disease caused by breast cancer gradually becomes larger. However, the etiology of breast cancer is far from fully understood. Great differences were found in tumor behavior, clinical manifestation, treatment response, and prognosis among different breast cancer types.^6^ Targeted therapy has become encouraging in breast cancer treatment, when estrogen receptor (ER) status was demonstrated to be an important treatment and prognostic factor in the last 10 years. Breast cancer is now considered a highly heterogeneous disease classified by distinct molecular profiles. Individualized treatment for breast cancer based on molecular pathology is becoming popular. In 2000, Perou et al^7^ for the first time proposed the concept of molecular subtype for breast cancer, including Luminal subtype, HER2 overexpression subtype, basal-like subtype, and normal-like subtype.

Compared with the western countries, China has a low incidence of breast cancer, but since the 1990s, its incidence has increased more than twice as fast as global rates, particularly in urban areas.^8^ Mortality from breast cancer rose progressively during the past 3 decades in both rural and urban areas, obviously differing from the international downtrend, which might partly be explained by improved quality of cancer registry data.

The patterns of breast cancer differ in many ways between Asian and Western countries.^9^ More attention was paid on the progress with time of epidemiology and clinicopathology of breast cancer. However, few articles were reported. Trend analysis is a technique that aims to identify a pattern of changes, or trend, in a series of observations. Therefore, we performed a retrospective study to understand the characteristics of breast cancer in Chinese patients and to identify the changes of epidemiology and clinicopathology in trends in 20 years from 1993 to 2013. The data would contribute much to understand the epidemiology of breast cancer in Southern China.

## METHODS

### Study Subjects

This was a retrospective study based on the data collected through hospital recorders from the General Hospital of the Chinese People's Liberation Army from January 1993 to September 2013. The patient diagnosed with breast cancer was recruited during this period. The following conditions were excluded: uncertain pathological diagnosis, complicated with other tumors, and death within 1 month of diagnosis. The research was approved by the Ethics Committee of the General Hospital of the Chinese People's Liberation Army. Patient consent was not required for this study because there were no anticipated risks for the participants of the study.

### Data Collection

The standard questionnaire was designed to collect the information through the medical recorder review. The information included: demographic characteristics, such as age and menstrual status at diagnosis, date of diagnosis, tumor side (left or right); pathological characteristics, such as tumor size, tumor type, tumor grade, lymph node status, ER and progesterone receptor (PR), and HER2 receptors status; and clinical features, such as stage at diagnosis and recurrent risk.

### Classification Criteria

Pathology type was based on the 1981, 2003, and 2012 WHO classification of tumors of the breast.^6,10,11^ Histology grade was based on the Nottingham Combined Histologic Grade, known as Elton-Ellis modification of Scarff-Bloom-Richardson grading system.^12^ Clinical staging of breast cancer was done according to the seventh edition AJCC Cancer Staging Manual.^13^ The evaluation of recurrence risk was based on the 2007 St. Galen Consensus.^14^ The classification of molecular subtype was based on the 2013 St. Galen Consensus.^15^

### Statistical Analysis

Means and standard deviations (SD) were described for the continuous variables with normal distribution and medians and ranges for the continuous variables with skewed distribution. An independent *t* test or a nonparametric test was used to determine the difference between groups, respectively. Frequencies and proportions were used for categorical variables. The differences were determined by a *χ*^2^ test or a Fisher exact test. The analysis of variance method was used to compare the differences in the multigroups. Trend test was used to detect the change trend of the related variables along with the time. A 2-sided *P* value of <0.05 was considered to be statistically significant. All analyses were performed using SAS software, version 9.1.3 (SAS Institute Inc, Cary, NC).

## RESULTS

### General Information

A total of 4968 patients with breast cancer were included from 1993 to 2013 in this study. The basic characteristics of patients were shown in Table 1. The mean ± SD of age was 49.5 ± 11.3 years and only 28 (0.6%) patients were male. Among all the 4968 patients, 1935 (39.2%) were postmenopausal women. Lesions in the left breast (2597, 52.3%) were slightly higher than the right breast (2255, 45.4%). The pathological type of patients was mainly invasive ductal carcinoma (3548, 71.4%), followed by mixed carcinoma (470, 9.5%). The patients were most diagnosed at early stage (stage II, 45.1% and stage I, 29.3%), totally accounting for 74.4%.

**TABLE 1:**
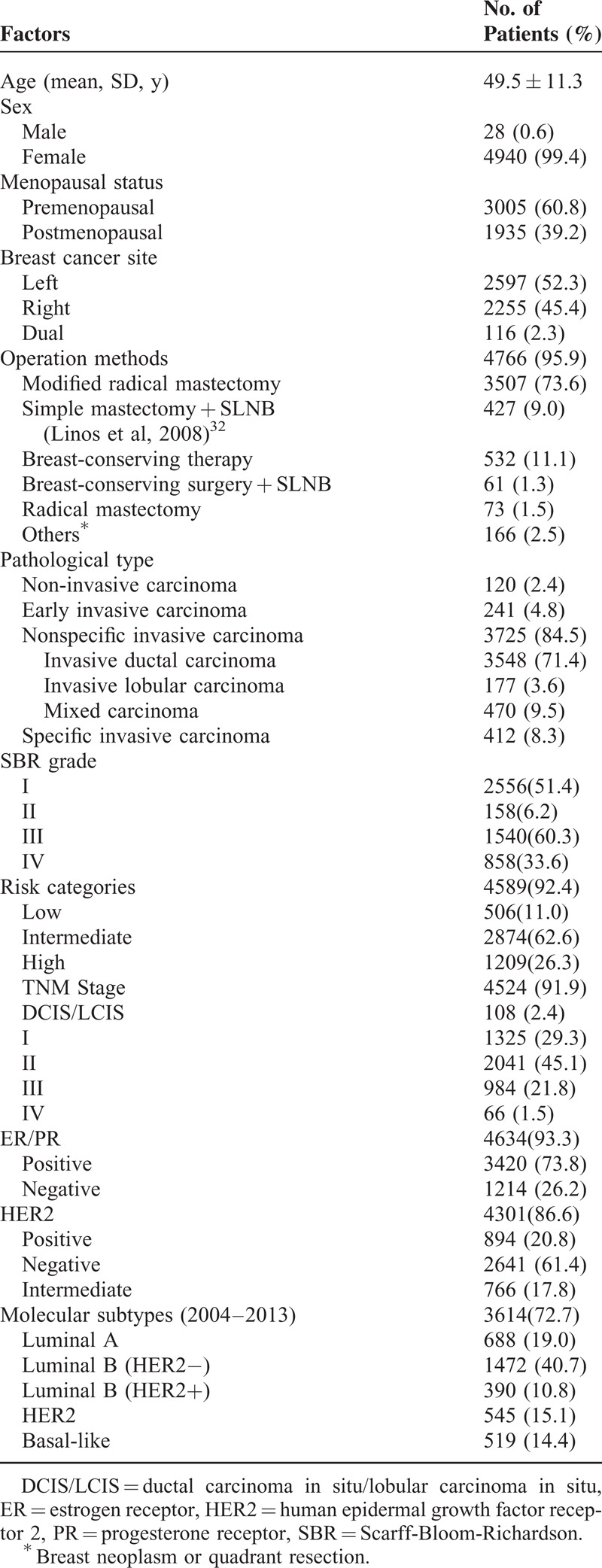
Basic Characteristics of Patients With Breast Cancer in the Study

### Age Dynamics of Patients

**FIGURE 1:**
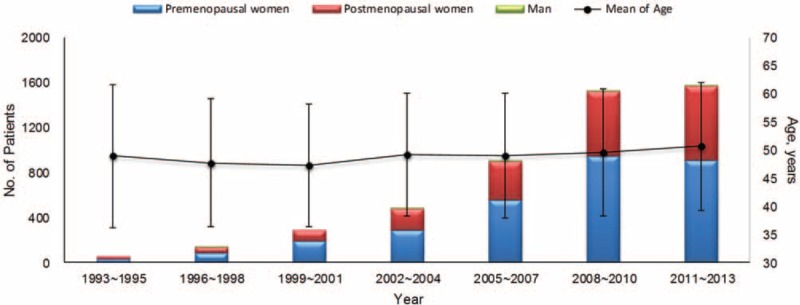
The distribution of patients numbers and mean age every 3 years from 1993 to 2013 in the study. The numbers of sex and status of menopause were also labeled in the figure.

The distribution and age dynamics of patients in every 3 years during 1993 to 2013 were demonstrated in Figure 1. The number of patients increased along with the year. The GLM analysis showed that the mean age at diagnosis also increased by 0.587 years per 3 years (*P* < 0.001). The mean ± SD age was 47.4 ± 11.3 years before the year 2001, 49.2 ± 11.2 years during 2001 to 2010 and 50.6 ± 11.4 years after the year of 2010, respectively (*P* < 0.001). While being classified into groups by every 5 years, the peak AAO (Age at Onset) of breast caner was 46 to 50 years (982, 19.8%), followed by 41 to 45 years (934, 18.8%) and 51 to 55 years (714, 14.4%), as well as 60 years or older (850, 17.2%), detailed information in supplemental Figure 1, http://links.lww.com/MD/A276. The proportion at age ≥60 years also increased from 14.0% to 19.3%; however, no obvious increase of the proportion at age ≥65 years was observed. The ratio of the premenopausal women to the postmenopausal women was 1.6 and no significant changes were found during the period (*P* = 0.121) in Figure 1.

### Pathological Features

**FIGURE 2:**
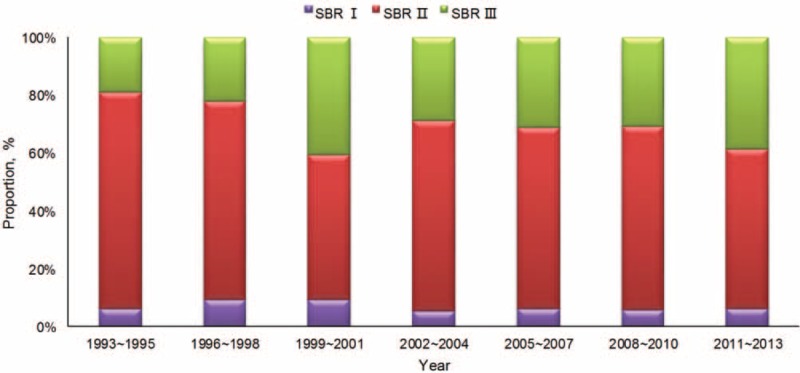
The proportions of SBR for patients with breast cancer in the study. SBR = Scarff Bloom Richardson.

The histological grades of the common pathological types among the patients with breast cancer were listed in Table 2. Scarff Bloom Richardson (SBR) II was the main grade in all the common pathological type except for the medullary carcinoma (SBR III). In tubular carcinoma and mucinous carcinoma, the proportions of SBR III were low (15.4% and 0%, respectively), whereas the proportions of SBR I were higher in other types of carcinoma (38.5% and 35.7%, respectively). The proportion of patients with SBR III breast cancer showed significant increase along with time (*P* = 0.015) (Figure 2), whereas the proportion of special types (except for invasive ductal carcinoma and invasive lobular carcinoma) decreased in the early years. The proportion of invasion of regional lymph node differed significantly among the common pathological types of breast cancer (*P* < 0.001) (Table 3). Compared with invasive ductal carcinoma and invasive lobular carcinoma, patients with mucinous carcinoma and tubular carcinoma were at lower risk of lymph node metastasis, whereas those with micropapillary carcinoma were at higher risk for lymph node metastasis, respectively.

**TABLE 2:**
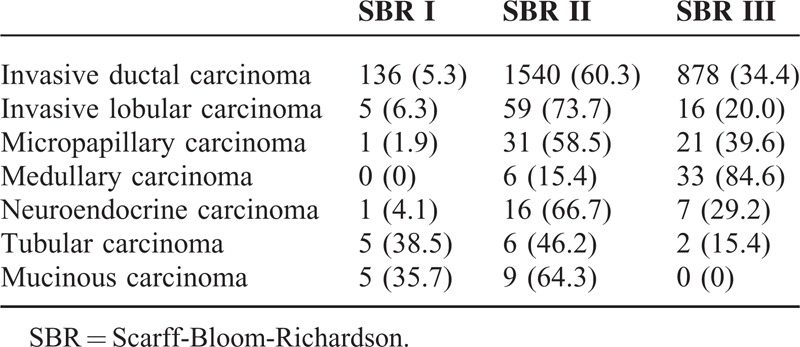
Histological Grades of the Common Pathological Types of Breast Cancer Among all the Patients

**TABLE 3:**
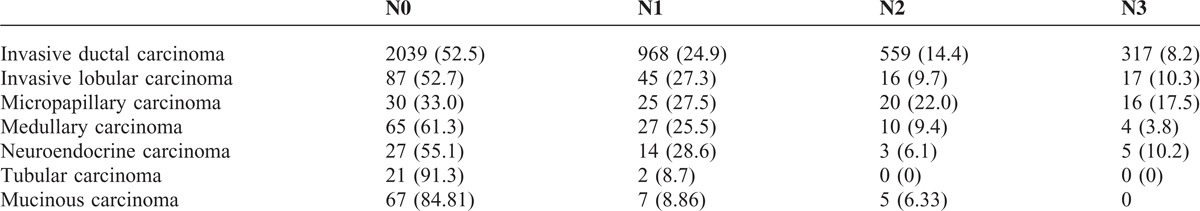
Regional Lymph Node Invasion of the Common Pathological Types of Breast Cancer Among all the Patients

### Clinical Features

**FIGURE 3:**
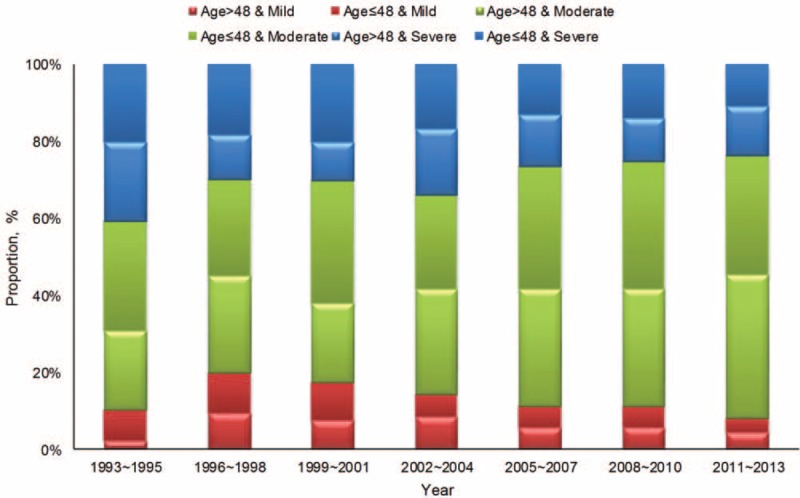
The distribution of disease risk of the patients with breast cancer in different periods.

**FIGURE 4:**
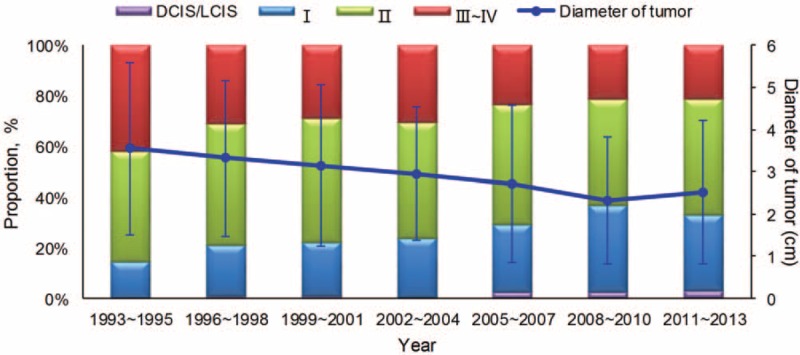
The distribution of clinical stage at diagnosis for breast cancer in the study.

The distribution of recurrent risk of the patients with breast cancer in different periods was shown in Figure 3. The results showed that the patients at the moderate risk tended to be more with time (*P* < 0.001), whether for patients ≥48 or <48 years old. The breast cancer at stage ductal carcinoma in situ/lobular carcinoma in situ (DCIS/LCIS) or I was accounting for 31.7% and the clinical stage at diagnosis gradually developed to early (*P* < 0.001). The proportion of DCIS/LCIS or stage I increased along with the time during the 20 years from 14.6% to 33.2%, whereas that of stage III to IV decreased (Figure 4), which were also found in the group of ≥48 years, <48 years and premenopausal women (all *P* < 0.001, Supplemental Figure 2, http://links.lww.com/MD/A276). It's different from that in the group of postmenopausal women (*P* = 0.060). It was also observed that the diameter of tumor got smaller at diagnosis.

### Molecular Features

**FIGURE 5:**
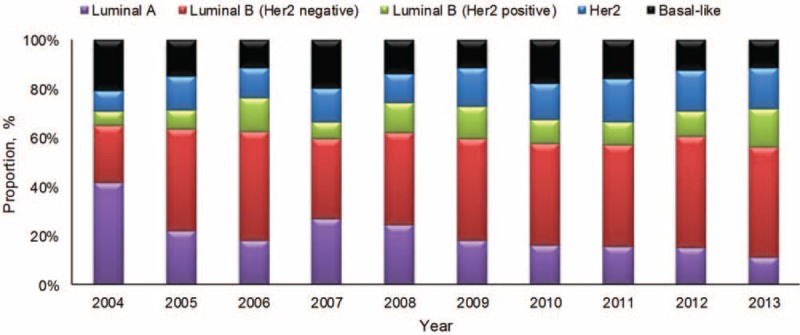
The distribution of molecular subtypes of breast cancer in the study from 2004 to 2013.

The positive rates of hormone receptor (HR) (ER/PR) and HER2 receptor were 73.8% (3420/4643) and 20.8% (894/4301), respectively. No significant time trends were presented for ER, PR, or HER2 receptor. Based on the rules of the St. Gallen International Expert Consensus (2013), the proportion of Luminal A type gradually reduced and Luminal B (HER2-negative) increased and developed to the predominant type (*P* < 0.001) (Figure 5). The former was observed in the 48 years or older group, whereas the latter in the older than 48 years group (Supplemental Figure 3, http://links.lww.com/MD/A276).

## DISCUSSION

Through analyzing the epidemiological and clinicopathological trends of breast cancer in China during the 20 years, we found a shift toward older age, an increasing proportion of early diagnosis, and the predominant trend of Luminal B (HER2-negative) in this study. This means aspects of breast cancer changed in many ways.

The global incidence of breast cancer has been increasing in recent years. In the developed countries, the incidence of breast cancer is over 100/100000, and over 9% of US women will have this disease in their life.^9^ China is also experiencing a rapid growth in breast cancer incidence. For the last 20 years, the average increase of breast cancer incidence in Beijing was 4.9%.^16^ The reported incidence of breast cancer for 2003 to 2007 was 68.6/100000 in Shanghai, 57.6/100000 in Beijing, 53.8/100000 in Guangzhou, and 45.9/100000 in Hong Kong.^17–19^

The mean age at diagnosis of breast cancer in China is 48 to 50 years, which is similar to other Asian countries and considerably younger than for Western women, for example 64 years in United States.^20–29^ In 2008, 16.6% of patients with breast cancer were aged 65 years or older in China (compared with 42.6% in the United States); 27.0% of patients with breast cancer in China are estimated to be 65 years or older by 2030.^30^ This was also been found in our study. The mean age at diagnosis increased nearly by 4 years during the 2 decades. On the basis of the growth rate calculated in our study, by 2030, the mean age will be reach 54 years. Of course, the proportion at 65 years or older will increase. Data from both Shanghai and Beijing show 2 age peaks, one at 45 to 55 years and the other at 70 to 74 years, with an increasing shift toward an older median age at diagnosis.^27^ Only 1 peak was revealed in our study: 40 to 50 years. It might be due to a birth cohort effect, resulting from changes in menstrual and reproductive patterns and other lifestyle and environmental factors that are prevalent in more recent birth cohorts.^31,32^ The peak at 70 to 74 years was not detected, which may be related to the patients in the early years.

A distinct difference in the menstrual status was noticed between Chinese patients and Western patients according to this study. Over half (60.8%) of our patients were premenopausal, whereas premenopausal women only account for 26% in global breast cancer patients. It is speculated that this is associated with the high prevalence of hormone replacement therapy in Western postmenopausal women.^27^

More frequent presentation with advanced-stage breast cancer is the main reason for disparities in survival between African American and white women in the United States.^33^ A multicenter nationwide study in China found that 15.7% of patients were diagnosed at stage I, 44.9% at stage II, 18.7% at stage III, and 2.4% at stage IV; the different stages were related to the socioeconomic status.^27^ In our study, the proportion was higher (29.3%) at stage I and lower at stage IV (1.5%). However, the proportion at stage IV was probably substantially underestimated in China, as most data are collected from surgical departments or inpatients, which caused Berkson bias. Howlader et al^34^ reported that 60% of women in the United States present with localized stage I and II disease, 33% present with regional stage III disease, and 5% present with stage IV disease. It is difficult to observe the differences among the groups, although nearly two-thirds of patients with breast cancer in China were diagnosed with advanced disease from a business survey,^35^ which was so large compared with other studies.

At present, no nationwide screening program for breast cancer was perfumed in China. Many factors, such as the widely dispersed population, inadequate insurance, insufficient convincing cost-effectiveness data, and so on, were barred to implementation of a population-based mammography screening program.^30^ However, the stage of breast cancer at diagnosis revealed earlier than before and the proportion of stage DCIS/LCIS and stage I has doubled during the 20 years observed from our study and the diameter of tumor has gotten smaller, which shows the progress in the early diagnosis of breast cancer. Self-examination probably improves awareness and might play a part in earlier-stage detection in China,^36^ as well as the better patient education, breast cancer screening, and primary medical care in the developed regions. Of course, public awareness also needs to be improved and more accessible health services should be provided for Chinese women willing to undergo screening to get benefits of earlier-stage detection.

Molecular subtypes such as HR and HER2 status are associated with patient prognosis and therefore affect the choice of treatment for breast cancer. In our study, HR was positive in 73.8% of the patients and HER2 receptor in 21% of the patients. These results are close to that of other Asian countries (HR: 57%–79%, HER2: 20%–25%). However, our patients had slightly lower HR-positive rate than in Europe (74% vs 84%) and slightly higher HER2 receptor-positive rate than in United States (21% vs 15%) compared with Western patients.^18,28,36–39^ The distribution of molecular subtypes and proportion of triple negative breast cancer were similar to that of other countries.^7,40^ The major molecular subtype was Luminal B (51.5%), the proportion of which kept increasing during the 20 years. This change was not caused by the evolution of the molecular classification standards. The subtypes of all the patients were defined by the St. Gallen International Expert Consensus (2013). Luminal B breast cancer has been reported to have lower expression of hormone receptors, higher expression of proliferation markers, and higher histologic grade than Luminal A. It also exhibits worse prognosis and has a distinct profile of response to hormone therapy and chemotherapy.^41^ So far, the molecular definition of Luminal B disease has also been of limited use in tailoring breast cancer treatment. Genotype-driven trials are already a reality in breast cancer research and will continue to contribute to the improvement of treatments for patients with Luminal B breast cancer.

## CONCLUSIONS

This study demonstrated the development, alternation, and progress of the epidemiology and clinicopathology of breast cancer in the past 20 years in China. Older age and earlier stage at diagnosis, as well as the alternation of predominant molecular subtypes, have become the developed trends of breast cancer.
